# Sex differences in the impact of frailty in elderly outpatients with heart failure

**DOI:** 10.3389/fcvm.2022.1000700

**Published:** 2022-09-12

**Authors:** Pablo Díez-Villanueva, César Jiménez-Méndez, Clara Bonanad, Carolina Ortiz-Cortés, Eduardo Barge-Caballero, Josebe Goirigolzarri, Alberto Esteban-Fernández, Angel Pérez-Rivera, Marta Cobo, Ancor Sanz-García, Francesc Formiga, Albert Ariza-Solé, Manuel Martínez-Sellés, Fernando Alfonso

**Affiliations:** ^1^Cardiology Department, Hospital Universitario de la Princesa, Universidad Autónoma de Madrid, CIBERCV, IIS-IP, Madrid, Spain; ^2^Cardiology Department, Hospital Clínico Universitario de Valencia, Instituto de Investigación Sanitaria (INCLIVA), Valencia, Spain; ^3^Cardiology Department, Hospital Universitario San Pedro de Alcántara, Cáceres, Spain; ^4^Cardiology Department, Complejo Hospitalario Universitario de a Coruña, A Coruña, Spain; ^5^Centro de Investigación Biomédica en Red de Enfermedades Cardiovasculares (CIBERCV), Madrid, Spain; ^6^Cardiology Department, Hospital Clínico Universitario San Carlos, Madrid, Spain; ^7^Cardiology Department, Hospital Universitario Severo Ochoa, Leganés, Spain; ^8^Cardiology Department, Hospital Universitario de Burgos, Universidad Isabel I, Burgos, Spain; ^9^Cardiology Department, Hospital Universitario Puerta de Hierro, Madrid, Spain; ^10^Unidad de Análisis de Datos, Instituto de Investigación Sanitaria del Hospital Universitario de la Princesa, Madrid, Spain; ^11^Servicio de Medicina Interna, Hospital Bellvitge, L'Hospitalet de Llobregat, Barcelona, Spain; ^12^Cardiology Department, Hospital Universitario de Bellvitge, L'Hospitalet de Llobregat, Barcelona, Spain; ^13^Cardiology Department, Hospital Universitario Gregorio Marañón, CIBERCV, Universidad Europea, Universidad Complutense, Madrid, Spain

**Keywords:** frailty, heart failure, elderly, sex, prognosis

## Abstract

**Introduction:**

Frailty is common among patients with heart failure (HF). Our aim was to address the role of frailty in the management and prognosis of elderly men and women with HF.

**Methods and results:**

Prospective multicenter registry that included 499 HF outpatients ≥75 years old. Mean age was 81.4 ± 4.3 years, and 193 (38%) were women. Compared with men, women were older (81.9 ± 4.3 vs. 81.0 ± 4.2 years, *p* = 0.03) and had higher left ventricular ejection fraction (46 vs. 40%, *p* < 0.001) and less ischemic heart disease (30 vs. 57%, *p* < 0.001). Women had a higher prevalence of frailty (22 vs. 10% with Clinical Frailty Scale, 34 vs. 15% with FRAIL, and 67% vs. 46% with the mobility visual scale, all *p*-values < 0.001) and other geriatric conditions (Barthel index ≤90: 14.9 vs. 6.2%, *p* = 0.003; malnutrition according to Mini Nutritional Assessment Short Formulary ≤11: 55% vs. 42%, *p* = 0.007; Pfeiffer cognitive test's errors: 1.6 ± 1.7 vs. 1.0 ± 1.6, *p* < 0.001; depression according to Yesavage test; *p* < 0.001) and lower comorbidity (Charlson index ≥4: 14.1% vs. 22.1%, *p* = 0.038). Women also showed worse self-reported quality of life (6.5 ± 2.1 vs. 6.9 ± 1.9, on a scale from 0 to 10, *p* = 0.012). In the univariate analysis, frailty was an independent predictor of mortality in men [Hazard ratio (HR) 3.18, 95% confidence interval (CI) 1.29–7.83, *p* = 0.012; HR 4.53, 95% CI 2.08–9.89, *p* < 0.001; and HR 2.61, 95% CI 1.23–5.43, *p* = 0.010, according to FRAIL, Clinical Frailty Scale, and visual mobility scale, respectively], but not in women. In the multivariable analysis, frailty identified by the visual mobility scale was an independent predictor of mortality (HR 1.95, 95% CI 1.04–3.67, *p* = 0.03) and mortality/readmission (HR 2.06, 95% CI 1.05–4.04, *p* = 0.03) in men.

**Conclusions:**

In elderly outpatients with HF frailty is more common in women than in men. However, frailty is only associated with mortality in men.

## Introduction

Heart failure (HF) is one of the main causes of morbimortality in older patients (1). Both its incidence and prevalence are increasing due, in part, to population aging (2, 3). However, elderly patients are still frequently underrepresented in clinical trials (4), and a better understanding of the clinical factors associated with prognosis in this population is needed (5). Frailty, which is common in elderly patients with HF, is an age-associated clinical syndrome characterized by a decrease in physiological reserve that entails an increased vulnerability to stressors (6–8). As such, frailty should be adequately both identified and addressed in HF patients (9).

Besides, sex-related differences in men and women with HF have been identified, not only from a pathophysiological point of view, but also regarding the different impact of traditional risk factors, together with specific sex-related factors and different prognosis in men and women (10, 11).

Our aim was to address the role of frailty and sex differences in the management and prognosis of elderly outpatients with HF.

## Methods

The FRAGIC registry (*impacto de la FRAGilidad y otros s*í*ndromes Geriátricos en el manejo cl*í*nico y pronóstico del paciente anciano ambulatorio con Insuficiencia Card*í*aca)* is an prospective observational multicenter study. The rationale of this study has been previously reported (12). Briefly, ambulatory patients ≥75 years with chronic HF treated according to current guidelines (13) were prospectively included between March and September 2019. Baseline clinical characteristics and laboratory and echocardiographic parameters were collected. Functional status and functional class as well as comorbidity and a systematic and comprehensive geriatric evaluation were registered in all patients at the first visit. Medical treatment was optimized according to clinical practice guidelines recommendations in all patients. Follow-up was carried out *via* clinical visit, electronic medical records review and/or telephone contacts at 1 year follow up. Total mortality and the need for hospitalization for any cause (duration >24 h) were recorded. The ethics committee of Hospital Universitario de La Princesa (Madrid, Spain) approved the study and the protocol was redacted according to the Declaration of Helsinki. All patients included in this study willingly completed the informed consent.

### Statistical analysis

For the purpose of this analysis, patients were divided by sex. Percentages were used to represent categorical variables, and the mean and standard deviation were used for continuous variables. The univariate comparison between each independent variable and sex, was assessed by Log-Rank test, from which *p*-values and Hazard ratios (HR) were obtained. Next, a predictive model was fitted using Cox Regression (multivariate analysis) by selecting those variables from the univariate analysis (*p* < 0.05 for women, and *p* < 0.001 for men, this difference is due to the high number of statistically significant variables in univariate analysis in men); this analysis was performed separately for women and men by considering as outcome mortality or the combination of mortality and readmission. Disease-specific survival or the cumulative event of readmission for any cause and mortality was obtained using the Kaplan-Meier method. Comparison of survival distributions was performed using a Log-Rank test. Data were analyzed using our own codes and basic functions in R, version 4.0.3 (http://www.R-project.org; the R Foundation for Statistical Computing, Vienna, Austria).

## Results

### Baseline characteristics and geriatric syndromes according to sex

A total of 499 ambulatory patients with chronic HF were included. Mean age was 81.4 ± 4.3 years, and 38% were women. Compared with men, women were older (81.9 ± 4.3 vs. 81.0 ± 4.2 years, *p* = 0.03) and had significantly higher left ventricular ejection fraction as well as less previous ischemic heart disease. Baseline variables are depicted in Table 1. Comorbidity prevalence was higher in men. Women had a higher prevalence of frailty and other geriatric conditions. Frailty was always more common in women irrespective of the scale (all *p* values< 0.001). Physical status according to short physical performance battery (SSPB) was also lower in women, in whom malnutrition and depression, as well as worse self-reported quality of life, were also more frequent (Table 1).

**Table 1:** Baseline characteristics.

	**Overall *n* = 499**	**Men (308, 61.7%)**	**Women (191, 38.3%)**	***p*-value**
Mean age (years)	81.4 ± 4.3	81.0 ± 4.2	81.9 ± 4.3	0.03
>85 years	25.9%	28.3%	24.3%	0.33
Body mass index (kg/m^2^)	27.6 ± 4.6	27.5 ± 4.1	27.7 ± 5.4	0.719
Hypertension	400 (80.3%)	241 (78.5%)	159 (83.2%)	0.238
Diabetes mellitus	199 (40%)	125 (40.7%)	74 (38.7%)	0.732
Dyslipidaemia	334 (67.3%)	210 (68.6%)	124 (65.3%)	0.498
Past smoker	166 (33.4%)	147 (47.9%)	19 (10%)	<0.001
Prior stroke	60 (12.1%)	38 (12.4%)	22 (11.5%)	0.758
Prior peripheral artery disease	55 (11.0%)	46 (14.9%)	9 (4.74%)	0.001
Atrial fibrillation	263 (52.7%)	163 (52.9%)	100 (52.4%)	0.975
Chronic obstructive pulmonary disease	74 (14.8%)	60 (19.5%)	14 (7.33%)	<0.001
Chronic renal failure	210 (42.1%)	128 (41.6%)	82 (42.9%)	0.835
Left ventricular ejection fraction (%)	42 ± 13	40 ± 12	46 ± 15	<0.001
NYHA ≥II	422 (84.5%)	255 (82.8%)	167 (87.4%)	0.106
Ischemic HF	161 (48.2%)	130 (56.8%)	31 (29.5%)	<0.001
Idiopathic HF	121 (36.2%)	70 (30.6%)	51 (48.6%)	<0.001
Systolic blood pressure (mmHg)	123 ± 19	122 ± 19	126 ± 19	0.028
Heart rate (bpm)	69 ± 12	69 ± 12	71 ± 12	0.027
**Laboratory findings**				
Hemoglobin (g/dl)	13.3 ± 1.7	13.7 ± 1.8	12.7 ± 1.4	<0.001
Platelet count (× 10^3^/mm^3^)	187 ± 54	179 ± 50	202 ± 59	<0.001
Leucocytes (× 10^3^/mm^3^)	7.1 ± 2.2	7.09 ± 2.0	7.07 ± 2.4	0.916
Lymphocytes (× 10^3^/mm^3^)	1.9 ± 1.3	1.87 ± 1.1	2.01 ± 1.5	0.254
Estimated glomerular filtration rate (eGFR, ml/min/1.72 m^2^)	52.1 ± 17.5	53.6 ± 17.4	49.6 ± 17.6	0.015
Sodium (mEq/L)	140 (3.1)	141 (3.1)	141 (3.1)	0.501
Potassium (mEq/L)	4.5 (0.5)	4.52 (0.5)	4.48 (0.5)	0.486
Brain natriuretic peptide NT proBNP (pg/ml)	2817 ± 3803	2940 ± 4032	2617 ± 3381	0.341
Ultrasensitive troponin (ng/ml)	26 ± 28	28 ± 32	20 ± 18	0.019
Cholesterol (mg/dl)	151 ± 35	145 ± 33	162 ± 36	<0.001
LDL-cholesterol (mg/dl)	80 ± 29	76 ± 27	88 ± 30	<0.001
Albumin (g/dl)	4.1 ± 0.4	4.1 ± 0.4	4.1 ± 0.4	0.514
Ferritin (ng/ml)	194 ± 18	200 ± 17	184 ± 22	0.424
Transferrin (mg/dl)	232 ± 47	229 ± 47	237 ± 48	0.126
Transferrin saturation (%)	24 ± 10	25 ± 10	23 ± 9	0.072
**Geriatric assessment and comorbidity**				
Comorbidity (Charlson index ≥4)	95 (19.0%)	68 (22.1%)	27 (14.1%)	0.038
Dependency (Barthel index ≤90)	96 (19.2%)	46 (14.9%)	50 (26.2%)	0.003
Dependency for daily activities (Lawton-Brody index ≤5)	183 (36.7%)	116 (37.7%)	67 (35.1%)	0.627
Pfeiffer cognitive test	1.22 ± 1.7	1.01 ± 1.6	1.57 ± 1.7	<0.001
Frailty (clinical frailty scale ≥4)	73 (14.6%)	32 (10.4%)	41 (21.5%)	0.001
Frailty (FRAIL)	111 (22.2%)	47 (15.3%)	64 (33.5%)	<0.001
Frailty (mobility visual scale ≥2)	269 (53.9%)	141 (45.8%)	128 (67.0%)	<0.001
Frailty (SPPB ≤9)	372 (74.5%)	211 (68.5%)	161 (84.3%)	<0.001
Nutrition status (MNA-SF ≤11)	235 (47.1%)	130 (42.2%)	105 (55.0%)	0.007
**Yesavage test**				
(v-15)	133 (26.6%)	59 (19.2%)	74 (38.7%)	<0.001
(v-5)	201 (40.3%)	101 (32.8%)	100 (52.4%)	
Self-reported quality of life (0–10)	6.8 ± 2	6.94 ± 1.91	6.47 ± 2.08	0.012
Average prescribed drugs	9.6 ± 3.2	9.6 ± 3.2	9.6 ± 3.3	0.93

### Clinical outcomes during follow-up according to sex

**Figure 1:**
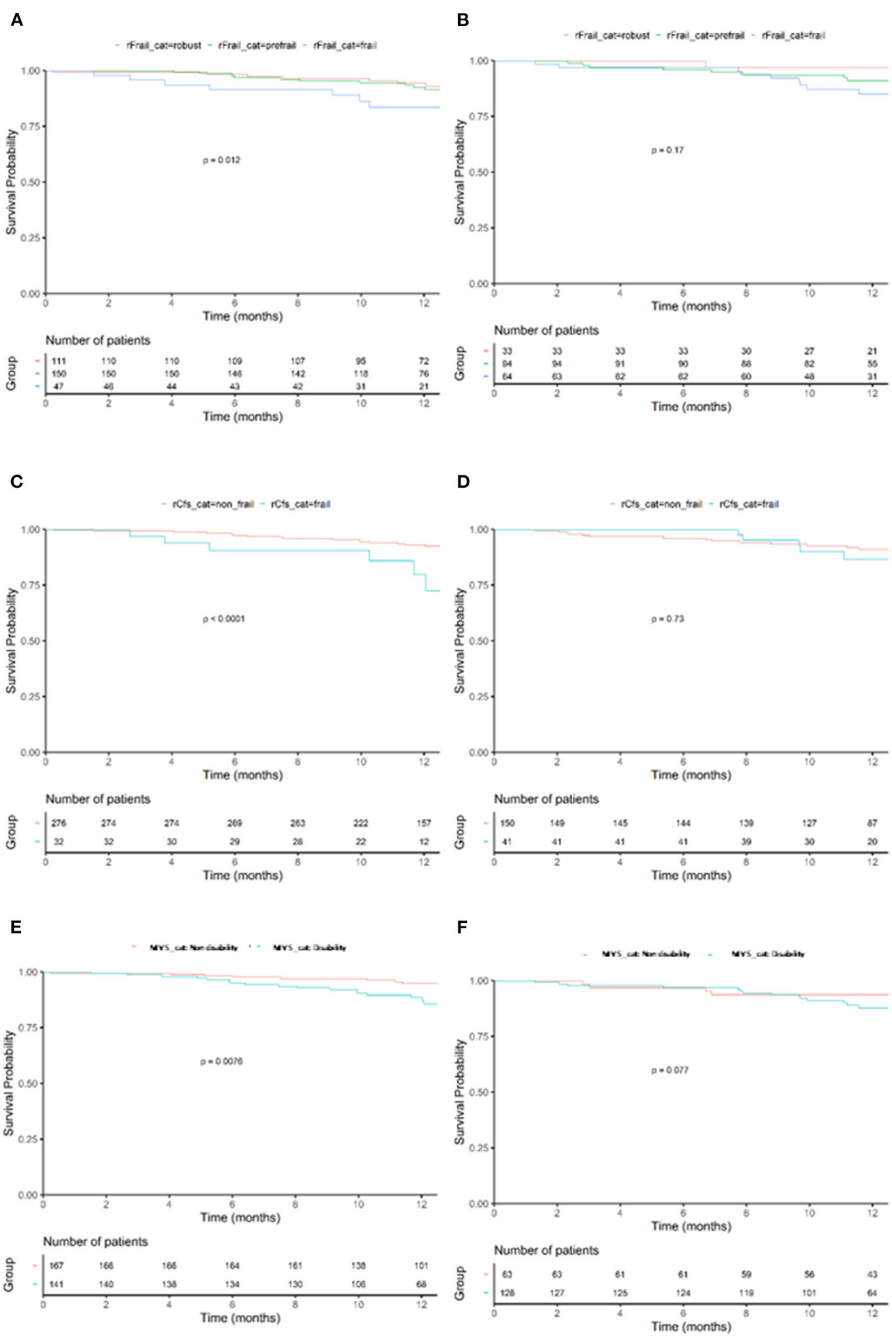
Kaplan–Meyer for 1-year mortality according to frailty category. Effects of frailty (FRAIL scale) in mortality in men **(A)** and women **(B)**. Effects of frailty (Clinical Frailty scale) in mortality in men **(C)** and women **(D)**. Effects of frailty (mobility visual scale) in mortality in men **(E)** and women **(F)**. *p*-value shows comparison by Log-Rank.

During a mean follow up of 371 (361–387) days, 58 patients (11.6%) died (32 men and 26 women). The leading cause of mortality was non-cardiovascular mortality (58%). Table 2 shows the variables associated with 1-year mortality according to sex in univariate analysis. In men, lower values of hemoglobin, lymphocytes, albumin and sodium, as well as urea and renal dysfunction were associated with mortality, whilst a lower platelet count was associated with prognosis in women. Data related to more advanced HF were associated with worse prognosis in women. Higher doses of diuretics, higher levels of natriuretic peptides and reduced right ventricular function were the only parameters independently associated with mortality in men and women. Frailty was associated with mortality only in men, although a trend toward higher mortality was observed in women according to some scales. Figure 1 shows the differential impact of frailty according to sex in mortality

**Table 2:** Variables significantly associated with 1-year mortality according to sex.

	**Men**				**Women**			
	**No event (*n* = 276)**	**Event (*n* = 32)**	**HR CI 95%**	***p*-value**	**No event (*n* = 165)**	**Event (*n* = 26)**	**HR CI 95%**	***p*-value**
Malignancy*	55 (19%)	13 (40.6%)	2.66 [1.31; 5.39]	0.007	28 (17.0%)	6 (23.1%)	1.51 [0.60; 3.76]	0.377
Hemoglobin (g/dl)*	13.9 (1.66)	12.1 (2.01)	0.56 [0.47; 0.68]	<0.001	12.8 (1.32)	12.3 (1.83)	0.77 [0.58; 1.02]	0.072
Platelets (× 10^3^ /μl)^†^	178 (48.0)	187 (64.8)	1.00 [1.00; 1.01]	0.331	205 (59.9)	184 (52.9)	0.99 [0.99; 1.00]	0.035
Lymphocites (× 10^3^/μl)*	1.92 (1.09)	1.42 (0.59)	0.38 [0.21; 0.69]	0.001	2.08 (1.65)	1.59 (0.76)	0.64 [0.40; 1.02]	0.058
eGFR (ml/min/1.72 m^2^)*	54.6 (17.1)	45.0 (17.3)	0.97 [0.95; 0.99]	0.002	49.8 (17.5)	48.3 (18.1)	0.99 [0.97; 1.02]	0.562
Urea (mg/dl)*	64.3 (30)	81.5 (42)	1.01 [1.00; 1.02]	0.002	66.9 (31)	77.1 (37)	1.01 [1.00; 1.02]	0.095
Sodium (mEq/L)*	141 (3)	139 (4)	0.81 [0.74; 0 0.91]	<0.001	141 (3)	141 (3)	1.00 [0.88; 1.13]	0.968
NT-proBNP (pg/ml)*^†^	2586 (3272)	6202 (7567)	1.00 [1.00; 1.00]	<0.001	2437 (3388)	3725 (3178)	1.00 [1.00; 1.00]	0.014
Albumin (mg/dl)*	4.15 (0.41)	3.92 (0.50)	0.27 [0.11; 0.65]	0.003	4.10 (0.42)	4.06 (0.44)	0.55 [0.16; 1.92]	0.348
Non-dilated right ventricle (%)^†^	224 (83.6%)	25 (80.6%)	0.80 [0.33; 1.95]	0.626	146 (89.6%)	12 (57.1%)	0.20 [0.08; 0.47]	<0.001
Systolic pulmonary artery pressure (mmHg)^†^	38.6 (11.8)	42.3 (12.2)	1.03 [1.00; 1.06]	0.072	38.8 (12.9)	49.8 (20.1)	1.03 [1.01; 1.05]	0.004
Significant tricuspid regurgitation^†^	29 (10.7%)	4 (12.9%)	1.45 [0.51; 4.16]	0.487	22 (13.6%)	11 (42.3%)	3.94 [1.80; 8.63]	0.001
Significant mitral regurgitation^†^	39 (14.3%)	8 (25.8%)	1.91 [0.85; 4.27]	0.116	18 (11.1%)	8 (30.8%)	4.31 [1.80; 10.3]	0.001
TAPSE (mm)*,^†^	18.3 (4.1)	16 (2.8)	0.88 [0.79; 0.97]	0.012	18.7 (3.7)	16.7 (4.3)	0.88 [0.79; 0.99]	0.032
Diuretic mean dose (mg of furosemide)*,^†^	55.1 (36.2)	80.8 (45.1)	1.01 [1.01; 1.02]	0.001	57.9 (32.3)	78.0 (45.8)	1.01 [1.00; 1.02]	0.004
Frailty (FRAIL)*	37 (13.4%)	10 (31.2%)	3.18 (1.29–7.83)	0.012	54 (32.7%)	10 (38.5%)	5.79 (0.74−45.3)	0.094
Frailty (CFS)*	23 (8.33%)	9 (28.1%)	4.53 (2.08–9.89)	<0.001	36 (21.8%)	5 (19.2%)	0.84 (0.32- 2.24)	0.732
Frailty (mobility visual scale ≥2)*	120 (43.5%)	21 (65.6%)	2.61 (1.26–5.43)	0.010	107 (64.8%)	21 (80.8%)	2.35 (0.88–6.24)	0.086
Malnutrition (MNA-SF ≤11)*	109 (39.5%)	21 (65.6%)	2.86 (1.38–5.94)	0.005	87 (52.7%)	18 (69.2%)	1.98 (0.86–4.57)	0.107

CFS, Clinical Frailty Scale; eGFR, estimated glomerular filtration rate; MNA-SF, Mini Nutritional Assessment Short Formulary; TAPSE, tricuspid annular plane systolic excursion.

^*^Variables significantly associated with 1-year mortality in men.

^†^Variables significantly associated with 1-year mortality in women.

**Figure 2:**
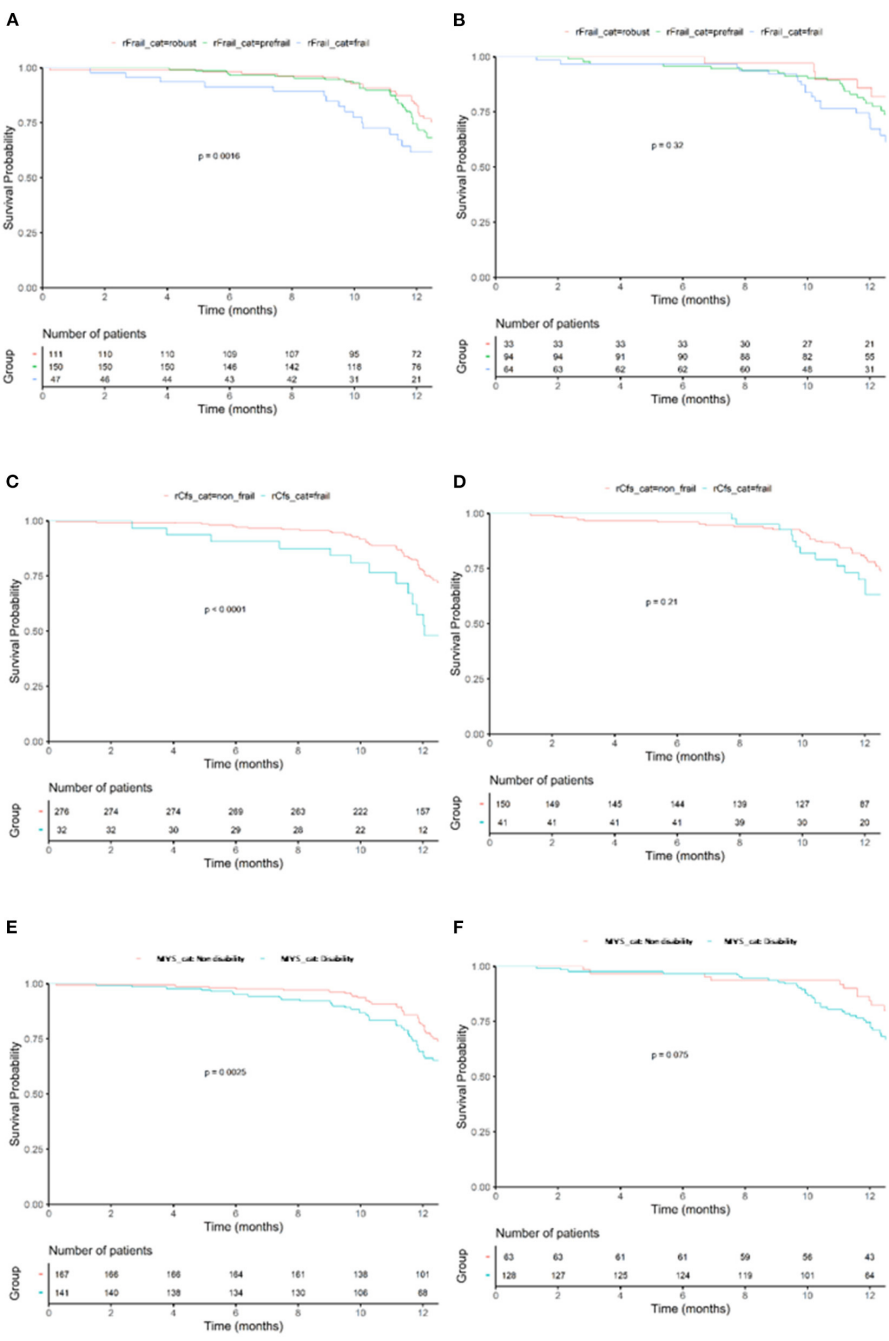
Kaplan–Meyer for 1-year mortality or readmission according to frailty category. Effects of frailty (FRAIL scale) in mortality or readmission in men **(A)** and women **(B)** Effects of frailty (Clinical Frailty scale) in mortality or readmission in men **(C)** and women **(D)**. Effects of frailty (mobility visual scale) in mortality or readmission in men **(E)** and women **(F)**. *p*-value shows comparison by Log-Rank.

During follow up, 202 patients (40%) fulfilled the 1-year composite endpoint of mortality and readmission for any cause:117 (38%) men and 85 (44.5%) women. Table 3 shows the variables associated with this endpoint according to sex in univariate analysis. Atrial fibrillation, physical signs of congestion, lower hemoglobin or lymphocytes levels, and a more advanced HF, were associated with mortality and readmission in men and women. Comorbidity and geriatric syndromes, were associated with worse prognosis in men, but not in women (except frailty estimated by Clinical Frailty Scale). Figure 2 shows the different impact of frailty in men and women in the composite endpoint.

**Table 3:** Variables significantly associated with 1-year mortality or readmission according to sex.

	**Men**				**Women**			
	**No event (*n* = 191)**	**Event (*n* = 117)**	**HR CI 95%**	***p*-value**	**No event (*n* = 106)**	**Event (*n* = 85)**	**HR CI 95%**	***p*-value**
Atrial fibrillation*,^†^	88 (46.1%)	75 (64.1%)	1.83 [1.25; 2.67]	0.002	50 (47.2%)	50 (58.8%)	1.62 [1.05; 2.51]	0.028
Number of previous HF admissions*	0.36 (0.59)	0.56 (0.87)	1.44 [1.15; 1.80]	0.001	0.53 (1.27)	0.56 (0.68)	1.02 [0.84; 1.23]	0.874
NYHA class ≥II*	150 (78.5%)	105 (89.7%)	2.07 [1.14; 3.77]	0.017	89 (84.0%)	78 (91.8%)	1.62 [0.75; 3.52]	0.220
Chronic pulmonary obstructive disease*	27 (14.1%)	33 (28.2%)	1.99 [1.33; 2.99]	0.001	6 (5.66%)	8 (9.41%)	1.30 [0.63; 2.69]	0.485
Chronic oxygen supply^†^	4 (2.09%)	3 (2.56%)	1.57 [0.50; 4.94]	0.444	4 (3.77%)	11 (12.9%)	1.99 [1.05; 3.75]	0.034
Peripheral artery disease*	19 (9.95%)	27 (23.1%)	1.87 [1.22; 2.88]	0.004	6 (5.71%)	3 (3.53%)	0.73 [0.23; 2.31]	0.593
Malignancy*	35 (18.3%)	33 (28.2%)	1.55 [1.04; 2.33]	0.032	18 (17.0%)	16 (18.8%)	1.08 [0.62; 1.86]	0.792
Peripheral congestion*	21 (11.1%)	26 (22.2%)	1.97 [1.28; 3.06]	0.002	13 (12.3%)	10 (11.8%)	1.08 [0.56; 2.10]	0.817
Pulmonary rales*,^†^	9 (4.74%)	14 (12.0%)	2.46 [1.40; 4.32]	0.002	2 (1.89%)	13 (15.3%)	4.13 [2.26; 7.52]	<0.001
Jugular venous distention*,^†^	6 (3.14%)	9 (7.69%)	2.19 [1.11; 4.33]	0.024	1 (0.95%)	6 (7.06%)	3.43 [1.47; 7.97]	0.004
Hemoglobin (g/dl)*,^†^	13.9 (1.63)	13.3 (1.97)	0.84 [0.76; 0.93]	0.001	12.9 (1.34)	12.6 (1.46)	0.86 [0.73; 1.00]	0.047
Lymphocites (× 10^3^/μl)*,^†^	1.97 (1.18)	1.70 (0.81)	0.70 [0.55; 0.91]	0.007	2.23 (1.90)	1.75 (0.97)	0.76 [0.60; 0.97]	0.025
Creatinine (mg/dl)*	1.30 (0.41)	1.46 (0.75)	1.39 [1.11; 1.73]	0.004	1.21 (0.80)	1.19 (0.41)	1.00 [0.72; 1.38]	0.994
Urea (mg/dl)*,^†^	62.8 (30.7)	71.2 (34.9)	1.01 [1.00; 1.01]	0.011	63.3 (28.0)	74.2 (36.5)	1.01 [1.00; 1.01]	0.030
Estimated glomerular filtration rate (eGFR, ml/min/1.72 m^2^)*	55.1 (16.7)	51.2 (18.2)	0.99 [0.98; 1.00]	0.030	50.4 (17.8)	48.7 (17.3)	1.00 [0.98; 1.01]	0.561
Sodium (mEq/L)*	141 (3.05)	140 (3.29)	0.92 [0.87; 0.98]	0.005	141 (3.27)	141 (2.95)	0.98 [0.92; 1.05]	0.647
Albumin (mg/dl)*	4.17 (0.38)	4.05 (0.48)	0.48 [0.29; 0.78]	0.003	4.11 (0.39)	4.09 (0.45)	0.68 [0.35; 1.30]	0.239
NT-proBNP (pg/ml)*	2356 (3092)	3911 (5102)	1.00 [1.00; 1.00]	<0.001	2686 (3993)	2536 (2487)	1.00 [1.00; 1.00]	0.781
Transferrin (mg/dl)*	235 (45.6)	219 (48.1)	0.99 [0.99; 1.00]	0.014	238 (45.1)	237 (51.9)	1.00 [0.99; 1.00]	0.798
Left ventricle hypertrophy*	84 (45.4%)	66 (58.4%)	1.67 [1.14; 2.43]	0.008	43 (41.7%)	39 (47.6%)	1.35 [0.87; 2.09]	0.178
Non-dilated right ventricle (%)^†^	157 (85.3%)	92 (80.0%)	0.71 [0.45; 1.12]	0.138	98 (94.2%)	60 (75.0%)	0.34 [0.21; 0.57]	<0.001
Systolic pulmonary artery pressure (mmHg)*,^†^	37.1 (11.0)	42.2 (12.7)	1.03 [1.01; 1.05]	0.001	37.7 (12.6)	43.9 (16.2)	1.02 [1.00; 1.03]	0.022
Significant tricuspid regurgitation^†^	21 (11.2%)	12 (10.4%)	1.07 [0.59; 1.95]	0.819	12 (11.5%)	21 (25.0%)	1.97 [1.20; 3.23]	0.008
Diuretic mean dose (mg of furosemide)*,^†^	50.7 (33.0)	67.4 (42.1)	1.01 [1.00; 1.01]	<0.001	53.8 (31.6)	69.3 (37.9)	1.01 [1.00; 1.01]	0.005
Comorbidity (Charlson index)*	3.03 (1.89)	3.80 (2.07)	1.16 [1.06; 1.26]	0.001	2.65 (1.59)	3.02 (1.85)	1.06 [0.94; 1.20]	0.319
Independency (Barthel index ≥90) *	168 (88.0%)	94 (80.3%)	0.59 [0.37; 0.93]	0.022	80 (75.5%)	61 (71.8%)	0.86 [0.54; 1.38]	0.540
Frailty (FRAIL)*	20 (10.5%)	27 (23.1%)	2.57 [1.55; 4.26]	<0.001	33 (31.1%)	31 (36.5%)	1.54 [0.79; 3.01]	0.201
Frailty (CFS ≥4)*,^†^	11 (5.76%)	21 (17.9%)	3.07 [1.91; 4.94]	<0.001	17 (16.0%)	24 (28.2%)	1.78 [1.11; 2.86]	0.018
Frailty (mobility visual scale ≥2)*	77 (40.3%)	64 (54.7%)	1.68 [1.17; 2.42]	0.005	68 (64.2%)	60 (70.6%)	1.22 [0.77; 1.95]	0.396
Depression*	55 (28.8%)	46 (39.3%)	1.50 [1.03; 2.17]	0.034	52 (49.1%)	48 (56.5%)	1.29 [0.84; 1.99]	0.239
Average prescribed drugs*	9.28 (3.09)	10.2 (3.31)	1.07 [1.01; 1.13]	0.015	9.27 (3.33)	10.1 (3.22)	1.05 [0.99; 1.11]	0.135

CFS, Clinical Frailty Scale; MNA-SF, Mini Nutritional Assessment Short Formulary.

*Variables significantly associated with 1-year mortality in men.

^†^Variables significantly associated with 1-year mortality in women.

In the multivariable analysis, frailty identified by the visual mobility scale was an independent predictor of mortality (HR 1.95, 95% CI 1.04–3.67, *p* = 0.03) and mortality/readmission (HR 2.06, 95% CI 1.05–4.04, *p* = 0.03) in men (Tables 4, 5). In women, higher doses of diuretics and higher levels of natriuretic peptides were the only factors significantly associated with mortality, while hemoglobin, right ventricular dilatation and higher diuretic doses were independently associated with mortality/readmission during follow-up (Tables 6, 7).

**Table 4:** Independent predictors of 1-year mortality in men.

	**HR**	**CI 95%**	***p*-value**
Hemoglobin (g/dl)	0.68	0.57–0.80	<0.001
Sodium (mEq/L)	0.94	0.86–1.02	0.17
NT-proBNP (pg/ml)	1.00	1.00–1.00	<0.001
Frailty (FRAIL)	1.45	0.81–2.59	0.20
Hemoglobin (g/dl)	0.67	0.56–0.79	<0.001
Sodium (mEq/L)	0.95	0.87–1.03	0.24
NT-proBNP (pg/ml)	1.00	1.00–1.00	<0.001
Frailty (CFS)	1.36	0.71–2.58	0.34
Hemoglobin (g/dl)	0.68	0.58–0.80	<0.001
Sodium (mEq/L)	0.94	0.87–1.03	0.21
NT-proBNP (pg/ml)	1.00	1.00–1.00	0.001
Frailty (mobility visual scale ≥2)	1.95	1.04–3.67	0.03

A Cox regression was performed for each of the different frailty scales to avoid collinearity between them: in each case, the significant variables (p < 0.001) were included in the log-rank test and the corresponding frailty scale.

**Table 5:** Independent predictors of 1-year mortality and readmission in men.

	**HR**	**CI 95%**	***p*-value**
NT-proBNP (pg/ml)	1.00	1.00–1.00	<0.001
Diuretic mean dose (mg of furosemide)	1.01	1.01–1.02	<0.001
Frailty (FRAIL)	1.59	0.86–2.95	0.13
NT-proBNP (pg/ml)	1.00	1.00–1.00	<0.001
Diuretic mean dose (mg of furosemide)	1.01	1.01–1.02	<0.001
Frailty (CFS ≥4)	1.99	0.95–4.14	0.06
NT-proBNP (pg/ml)	1.00	1.00–1.00	0.002
Diuretic mean dose (mg of furosemide)	1.01	1.01–1.02	<0.001
Frailty (mobility visual scale ≥2)	2.06	1.05–4.04	0.03

CFS, Clinical Frailty Scale.

A Cox regression was performed for each of the different frailty scales to avoid collinearity between them: in each case, the significant variables (p < 0.001) were included in the log-rank test and the corresponding frailty scale.

**Table 6:** Independent predictors of 1-year mortality in women.

	**HR**	**CI 95%**	***p*-value**
Platelets (× 10^3^/μl)	1.00	0.99–1.00	0.91
NT-proBNP (pg/ml)	1.00	1.00–1.00	<0.001
TAPSE (mm)	0.96	0.87–1.06	0.45
Non-dilated right ventricle (%)	0.75	0.32–1.75	0.51
Systolic pulmonary artery pressure (mmHg)	1.02	0.99–1.04	0.09
Diuretic mean dose (mg of furosemide)	1.01	1.00–1.01	0.04
Significant mitral regurgitation	1.26	0.56–2.78	0.56
Significant tricuspid regurgitation	1.39	0.55–3.55	0.48

TAPSE, tricuspid annular plane systolic excursion.

**Table 7:** Independent predictors of 1-year mortality and readmission in women.

	**HR**	**CI 95%**	***p*-value**
Atrial fibrillation	0.81	0.38–1.71	0.58
Chronic oxygen supply	0.69	0.17–2.72	0.59
Pulmonary rales	1.43	0.41–5.00	0.56
Jugular venous distention	1.62	0.47–5.55	0.43
Hemoglobin (g/dl)	0.76	0.60–0.97	0.03
Lymphocites (× 10^3^/μl)	0.68	0.46–1.01	0.05
Urea (mg/dl)	0.99	0.98–1.00	0.47
Non-dilated right ventricle (%)	0.45	0.20–0.99	0.04
Systolic pulmonary artery pressure (mmHg)	1.01	0.98–1.03	0.35
Diuretic mean dose (mg of furosemide)	1.01	1.00–1.01	0.04
Frailty (CFS ≥4)	1.46	0.56–3.79	0.43
Significant tricuspid regurgitation	1.55	0.69–3.48	0.28

CFS, Clinical Frailty Scale.

## Discussion

To the best of our knowledge, this is the first study addressing sex differences in the impact of frailty in elderly ambulatory patients with chronic HF followed by cardiologists. Main findings of our study are: (1) elderly men and women with chronic HF show a different baseline and clinical profile; (2) frailty and other geriatric syndromes are more common in women, although they only associate worse prognosis in men; (3) some parameters common in advanced stages of HF entail worse prognosis in men and women, but differ between them.

There are several sex differences in patients with HF previously reported, as traditional risk factors, pathophysiology and response to treatment differs between men and women (10, 11, 14, 15). In a large multicentre study, including >80,000 hospitalized patients, Hsich et al. described, more than a decade ago, that women with HF were usually older than men, more likely to have hypertension and depression and less likely to have coronary or peripheral artery disease. However, in-hospital mortality rates were similar irrespective of sex (16). Our study showed similar results, since HF women were older, and had less frequently a previous history of coronary or peripheral artery disease. However, patients included in our study were all ambulatory patients with chronic HF (i.e., not hospitalized), and mean age was much higher. Besides, our study adds novel evidence with valuable data from the late clinical follow up, unlike the study by Hsich et al.

In FRAGIC study, women presented with better LVEF compared with men, as previously reported (10). Such differences regarding the subtype of HF have been suggested to be partially explained due to inherent physiological distinctions between men and women (17, 18). Regarding clinical presentation, some studies suggest women usually present with worse functional class and more advanced symptoms (10, 11, 14). Interestingly, in our study key issues like NTproBNP levels or NYHA functional class did not differ at baseline between men and women, unlike other previous studies, in which female sex had been associated with worse functional class and even higher NTproBNP levels regardless of LVEF (14, 19). In FRAGIC study, higher levels of natriuretic peptides and diuretics doses were significantly associated with higher mortality in women at 1 year follow-up. On the other hand, lower hemoglobin and sodium levels and higher NTproBNP levels independently associated poorer prognosis in men, together with the presence of frailty identified by the visual mobility scale.

Regarding geriatric conditions, HF commonly coexists with frailty, especially in the elder population, yet the prevalence of frailty varies according to the scale used. Both conditions when present together lead to worse outcomes (3, 20). Thus, it is recommended to properly assess its presence (9, 21), since the greater accumulation of deficits in frailty domains, the greater the mortality (22). Notably, frailty affects women significantly more than men in HF, as demonstrated in a recent meta-analysis including 29 studies, in which the relative risk of frailty was found to be 26% higher in women compared with men (23). As expected, the relative risk of frailty in women was higher when defined with a physical approach. In this regard, Denfeld et al. performed a small prospective single-center study (including 115 patients, mean age 63.6 ± 15.7 years, 49% women) aimed to characterize sex differences in physical frailty in HF. Authors found that women with HF were significantly more likely to be physically frail than men. Frailty was related with higher overall comorbidity burden in both men and women although frail women had a worse symptom profile (24). However, such population was significantly younger than that in our study (mean age 63.6 vs. 81.4 years) and had different baseline characteristics: 71% had reduced LVEF and almost 50% had NYHA III-IV functional status (which may, in part, explain the discrepant findings). In our study, women were more commonly frail than men, irrespective of the scale. Hence, it could be hypothesized that these differences may rely on the fact that frailty scales might not adequately identify (or even overestimate) the presence of frailty in women. However, the FRAIL scale was developed in a cohort of 4,000 patients, 50% women and this scale was later validated in a mostly-women community population (25, 26). FRAIL scale has been also validated in a sample of 703 patients, 40% women (27), whereas the CFS was developed in a prospective cohort of 2305 patients, 61% women, from the Canadian Study of Health and Aging (CSHA) (28). Interestingly, in our study, women showed significantly worse self-reported quality of life. This finding has been previously reported in some studies, closely related to HF status (10), though it has also been found to be higher in frail patients (Souza).

Concerning the prognosis of frailty in HF patients, a recent metanalysis showed it was associated with an approximately 1.5-fold increase risk of death and hospitalization in HF patients, although differences between men and women were not explored (29). However, results of this study should be taken with caution, since the sample had high heterogeneity, with some studies including patients during an acute HF episode, and frailty was not uniformly defined. On the other hand, in a recent study including nearly 600 patients admitted with decompensated HF (mean age 76.6 years, 45% women), patients with higher CFS score showed a worse clinical profile and had higher probability of all-cause death and rehospitalisation in both men and women (30). Besides, it is recommended to assess frailty in an ambulatory fashion, and not in the setting of an acute HF event, as in those studies (9). In our study, frailty identified by the mobility visual scale was independently associated with mortality/readmission in men.

Recently, St Sauver et al. (31), demonstrated the negative relationship between inflammation, multi-morbidity and biologic aging, in such a way that men and elderly people, especially with higher comorbidity, had significantly higher levels of inflammatory biomarkers. This, in turn, has been linked with the concept of “*inflammaging*,” key component of the aging process. Soysal et al. (32) have associated this concept with the development of cardiovascular disease and frailty. Thus, although the prevalence of frailty was lower in men in our study, it could be hypothesized that a greater proinflammatory state might explain, at least in part, why it had a greater prognostic impact in older men with heart failure.

Our study, despite its prospective design, has some limitations that merit discussion. First, it is an observational study so we cannot rule out the possibility of selection bias. On the other hand, the sample size was modest, and the percentage of women included lower than that in other similar studies. Also, the 1-year event rate was relatively low, therefore results should be extrapolated with caution, particularly to other settings, since our study only included elderly ambulatory patients with chronic HF followed by cardiologists. In spite of these limitations, we think that this study provides new and interesting information on gender differences in the impact of frail in older patients from a large cohort of consecutive unselected elderly HF patients. Further studies will be required to elucidate the underlying reasons explaining a distinct effect of frailty according to gender.

## Conclusion

Elderly women with HF present frailty and other geriatric conditions more often than men, although frailty is only associated with worse prognosis in men.

## Data availability statement

The original contributions presented in the study are included in the article/supplementary material, further inquiries can be directed to the corresponding author.

## Ethics statement

The studies involving human participants were reviewed and approved by CEIm Hospital Universitario de La Princesa, Madrid, Spain. The patients/participants provided their written informed consent to participate in this study.

## Author contributions

PD-V and CJ-M prepared the first draft of the manuscript. All authors improved the manuscript with relevant content, contributed to the article, and approved the submitted version.

## Conflict of interest

The authors declare that the research was conducted in the absence of any commercial or financial relationships that could be construed as a potential conflict of interest.

## Publisher's note

All claims expressed in this article are solely those of the authors and do not necessarily represent those of their affiliated organizations, or those of the publisher, the editors and the reviewers. Any product that may be evaluated in this article, or claim that may be made by its manufacturer, is not guaranteed or endorsed by the publisher.
